# Testing feedback and social norms messages to increase participation: the BESTcc randomised controlled trial among non-respondents in population-based colorectal cancer screening programmes in Tuscany

**DOI:** 10.1016/j.ssmph.2026.101974

**Published:** 2026-09-22

**Authors:** Giuseppe Gorini, Elisa Betti, Patrizia Falini, Giulia Carreras, Beatrice Mallardi, Manola Migliori, Daniela Giorgi, Daniela Tramalloni, Alessandro Cosimi, Giulia Venturi, Andrea Amelio Ravelli, Anna Iossa, Francesca Battisti, Sandro Tiziano Stoffel, Paola Mantellini

**Affiliations:** a Istituto per lo Studio la Prevenzione e la Rete Oncologica (ISPRO), Italy; b Azienda USL Toscana Nord Ovest, Italy; c Azienda USL Toscana Centro, Italy; d Azienda USL Toscana Sud Est, Italy; e Institute for Computational Linguistics “A. Zampolli” (CNR-ILC) Pisa, Italy; f Research Department of Behavioural Science and Health, University College London, London, UK; g Institute of Pharmaceutical Medicine, University of Basel, Basel, Switzerland

**Keywords:** Colorectal cancer screening, Trial, Behavioural economics, Social norms, Feedback

## Abstract

• Trial embedded in routine CRC screening for non-responders in Tuscany.
• RCT tested social norms and feedback to boost FIT screening uptake.
• All intervention arms improved FIT return vs standard invitation.
• Combined messages offered no added benefit over single interventions.

## 1 Introduction

Colorectal cancer (CRC) is a leading cause of cancer deaths worldwide (Xi & Xu, 2021). Early detection with faecal tests (FOBT/FIT) reduces mortality (Frazier et al., 2000), but screening effectiveness depends on high uptake (Gimeno Garcia, 2012). European guidelines set a minimum of 45% and a desirable 65% (Segnan et al., 2010). Reported participation for faecal tests ranges from 23% to 63% with socioeconomic gaps, (Basu et al., 2018; Hirst et al., 2018; Klabunde et al., 2015). In Italy, the 2023 national participation rates averaged 34.9% (range 18.9% in Lazio to 64.2% in Veneto), with Tuscany at 45.8%, below the desirable target (Costantini et al., 2008; Osservatorio Nazionale Screening, 2025; Zorzi et al., 2008).

Efforts to enhance participation initially focused on rational, reflective decision-making (System 2 thinking) (Purnell et al., 2015; Taylor et al., 2022). According to dual-process theory, behaviour is shaped by two interacting systems: a fast, automatic System 1 and a slower, deliberative System 2 (Frankish, 2010). Recently, interventions inspired by behavioural economics (BE), which target intuitive, automatic decision-making (System 1 thinking), have emerged. These interventions leverage heuristics, automatic learned responses, through small contextual modifications, known as ‘nudges’ (Dolan et al., 2012; Thaler & Sunstein, 2009). Examples include adjusted decision settings, financial incentives, feedback on past behaviour, or social norms messages (Purnell et al., 2015; Taylor et al., 2022). Feedback and social norms try to encourage people to align their behaviour with that of their peers.

Social norms, whether descriptive (how others behave) or injunctive (expected behaviour), offer a behavioural standard individuals may be motivated to follow (Cialdini & Trost, 1998; Schultz et al., 2007). While social norms have shown positive impacts on various health behaviours (Sheeran et al., 2016), outcomes for CRC screening remain unclear (Bakr et al., 2020; Gorini et al., 2023, 2026; Stoffel et al., 2019, 2021).

Similar to social norms, feedback on past behaviour serves as a nudge by increasing individuals' awareness of their behavioural patterns, thereby promoting positive change. This approach often emphasizes discrepancies between previous actions and desired outcomes or societal norms, enhancing self-awareness and motivating individuals to align their behaviour with recommended practices (Goldstein et al., 2008). Feedback interventions have been successfully applied to influence energy consumption, physical activity, and CRC screening participation (Darby, 2006; Godino et al., 2013; Normand, 2008). Both approaches act on similar automatic-motivational pathways and could, in principle, be combined to enhance effects (Godino et al., 2013; Sheeran et al., 2016). Two recent studies on CRC screening, however, found no added benefit from combining feedback with social norms messages (Gorini et al., 2023, 2026).

The trials tested adding feedback, a minority-norms message, or both to invitation letters sent to previous non-responders (Gorini et al., 2023, 2026). In one trial of over 11,000 invitees from Florence, Rome, and Turin, all three modified invitation letters increased uptake (7.0%–8.2% vs. 5.3% for the standard letter) (Gorini et al., 2023). The minority-norms message was particularly effective among individuals aged 54–64, whereas feedback, alone or combined with social norms, improved participation among those aged 60–64. None of the interventions affected participation among individuals over 65. Differences across the three screening programmes, such as Turin contributing only 4.1% of invitees (mostly older adults), may have influenced these results.

The second trial in Florence, involving 20,225 non-responders, found that feedback increased uptake to 7.4%, while the social norms (6.7%) and combined messages (6.6%) did not yield statistically significant improvements compared to the control condition (5.7%) (Gorini et al., 2026).

In this study, we extend previous research on social norms and feedback messages by testing them in Tuscany, a region that includes both urban and rural screening organizations. As in the earlier two trials, the work was conducted within the BESTcc project, which evaluates new interventions to improve participation in Tuscany's CRC screening programmes. (ISRCTN11841256)

## 2 Methods

### 2.1 Study design

The randomised controlled trial (RCT) employed a 2×2 factorial experimental design, replicating the structure of our two previous studies (Gorini et al., 2023; Stoffel et al., 2021), with two factors under investigation: inclusion of a minority-norms message and inclusion of a feedback message. Previous non-attenders were randomly assigned to one of four study arms:

1. a control group receiving the standard invitation letter;
2. a feedback group receiving the standard letter including a feedback message (F);
3. a social norms group receiving the standard letter including a minority-norms message (MN);
4. a combined group receiving the standard letter including both the feedback and minority-norms messages (F + MN).

A second invitation was sent to individuals who had not completed screening within 45 days. The primary objective of this study was to evaluate the effectiveness of these BE messages in increasing CRC screening participation measured by return of FIT test within 90 days after the first invitation.

The trial was registered with the ISRCTN registry in 2022 (ISRCTN11841256). The study protocol was approved by the Ethics Committees of the Local Health Units in Tuscany (Comitato Etico Toscana Area Vasta Centro, ID: 16875_spe, approved 19/01/21). Individual informed consent was not collected, as the trial was integrated into routine CRC screening, an Essential Level of Care (LEA) in the Italian National Health System, and it aimed to evaluate the effectiveness of the experimental invitation messages.

### 2.2 Participants

The RCT was conducted in ten of the twelve CRC screening programmes implemented within the three Local Health Units (LHUs) of the Tuscany region: North-Western Tuscany LHU (covering Massa Carrara, Viareggio, Lucca, Pisa, and Livorno), South-Eastern Tuscany LHU (Siena and Grosseto), and Central Tuscany LHU (Florence, Pistoia, and Prato). All programmes invite residents aged 50–70 to undergo a FIT test every two years. The study targeted individuals who had not responded to screening invitations on at least two consecutive occasions and had never been screened in these programmes. These were all aged between 54-70. Individuals not meeting the inclusion criteria were excluded. Specifically, exclusion criteria comprised a personal history of CRC, adenomas, or inflammatory bowel diseases; a colonoscopy within the past 5 years; or a faecal occult blood test within the past 2 years. Individuals with invalid mailing addresses were included in the intention-to-treat (ITT) analysis.

### 2.3 Randomisation

Individuals were individually randomised using a pseudo-random number generator in a 3:1:1:1 ratio. We used a 3:1 control-to-intervention ratio to ensure enough participants in the experimental arms while avoiding major disruption to the ongoing CRC screening program. Individuals were assigned to receive either a standard invitation letter (control) or one of three modified letters incorporating BE-inspired messages: Feedback (F), Minority Norms (MN), or a combination of Feedback and Minority Norms (F + MN). Due to the nature of the intervention, neither researchers nor participants were blinded to group allocation. However, participants were unaware they were part of a research trial, and staff involved in enrolment and assignment were blinded to the allocation sequence.

### 2.4 Messages

The behavioural messages used in this trial were identical to those used in our previous two studies, as they were co-developed and pretested with public and patient representatives through qualitative focus groups (Gorini et al., 2023, 2026). Table 1 provides the English translations of the three BE-inspired messages, which were incorporated into the second paragraph of the invitation letter. The feedback message informed non-attenders that, unfortunately, they had not participated previously. The sentence aimed to raise awareness of their past behaviour while emphasizing the importance and desirability of future screening (Allcott, 2011).

**Table 1:** Behavioural messages used in the Trial (translated from Italian).

	Message
Feedback	Unfortunately, you have not responded to previous invitations. However, you should know that you can still do so. We hope that you can participate this time because it is never too late to take care of your health by preventing bowel cancer.
Minority norms	In many areas, 6 out of 10 people invited to participate in the screening program accept the invitation. You are part of the minority who do not participate. We hope that this time you will join the group of people who respond to our invitation because it is never too late to take care of your health by doing bowel cancer prevention.
Feedback + Minority norms	In many areas, 6 out of 10 people invited to participate in the prevention program accept the invitation. You are in the minority who do not participate. Unfortunately, you have not responded to previous invitations, but we hope that this time you will join the group of people who respond to our invitation because it is never too late to take care of your health by doing bowel cancer prevention.

The social norms message informed individuals that, in many areas, 6 out of 10 invitees had participated in screening already and that the recipient was among the minority who had not. This message sought to address misperceptions by emphasizing majority peer participation, thereby motivating non-attenders to engage in CRC screening (Allcott, 2011; Schultz et al., 2007; Sieverding et al., 2010). In the combined condition, the message presented the social norms information first, followed by the feedback. In all three conditions, a concluding sentence emphasized that individuals could still participate in the screening by responding to the invitation.

We note that the social norms message contains an implicit feedback element, since it informs recipients that they are among the minority who did not participate.

To assess whether the added messages compromised linguistic clarity, we evaluated their complexity using automated readability assessment tools based on natural language processing (NLP) techniques (Dell’Orletta et al., 2014). These results were validated through crowd-sourced comprehension testing, aimed at verifying whether human readers perceived the messages as comparatively more linguistically complex after the modifications.

### 2.5 Outcomes

The primary outcome was FIT return rate, defined as the percentage of individuals returning a faecal sample within 90 days following the first invitation. Samples returned after 90 days were considered non-responses. Screening participation across experimental conditions was compared using an ITT analysis, which included individuals whose invitation letters were undelivered and returned to the screening centres. FIT return rate was stratified according to local health units.

### 2.6 Statistical analysis

The sample size was calculated a priori to detect a minimum difference of 3 percentage points in participation between the experimental (Feedback or the Minority Norms arms) and control conditions, with 80% statistical power and a significance level (α) of 0.05.

In the 2x2 factorial design analysis, we analysed the main effect of feedback (present vs absent), the main effect of minority norms, and their interaction (Odds Ratio: 0.80; 95%CI: 0.72-0.90). Considering that the interaction was significant (likelihood ratio test p-value = 0.0001), we stratified results by modality: Feedback vs. Controls; Minority Norms vs. Controls; Feedback + Minority Norms vs. controls.

To evaluate the impact of the three BE-inspired messages across the 10 screening programmes, we implemented a mixed-effects logistic regression model to assess the effect of study arm on participation. To account for the correlation among individuals within the same screening program — in terms of both individual characteristics and screening compliance — the probability of screening participation in program j (j from 1 to 10) by individual i, P(Yij = 1), was modelled by means of a random intercept and random coefficient logit model:

logit[P(Yij = 1)] = b0j + b1j xij, b0j = b0+u0j, b1j = b1+u1j

with eij normally distributed individual error term and u0j and u1j random variables with a bivariate Normal distribution in the population of screening program. A likelihood ratio test confirmed the superiority of the multilevel model over a single-level one (χ^2^(3) = 671.58, p < 0.0001), and further comparison favoured the model with random intercept and slope over one with a random intercept only (p = 0.0302). The variance of the slope was 0.0034 (95%CI: 0.0007 – 0.1570), whereas the variance of the intercept was 0.3189 (95%CI: 0.1196 – 0.8508), that is 98.9% of the total variance. The Intraclass Correlation for the variable of the screening programmes was 0.0884 (95%CI: 0.0351 - 0.2055). The interaction analysis was pre-specified in the study protocol, and we tested interaction between study arm and age or gender as possible interaction variables. Due to a significant interaction between study arm and age group (p = 0.0362), the model was stratified by age group and adjusted for gender. Then, we calculated Absolute Risk Reduction (ARR), which is the difference between the participation rates in control and experimental arms, and the Number Needed to Treat (NNT), which is the number of patients who need to be invited using a specific behavioural message to get one more participant. Statistical analyses were conducted using Stata/SE version 16.1 (StataCorp LP, College Station, TX). No data were missing.

### Role of the funding source

The funders of this study had no role in study design, data collection, data analysis, data interpretation, or the writing of the report.

## 3 Results

### 3.1 Study sample

Between January 2022 and April 2024, 85,193 study participants were randomised across four conditions (see Fig. 1 and Table 2). Most were aged between 60 and 70 years (N = 47,063, 55.2%), male (N = 43,520, 51.0%), and recruited from screening programmes in Central Tuscany (N = 35,422, 41.6%). Differences in the distribution of individuals among the four arms were observed regarding age group: a higher proportion of individuals aged 60–70 years were in the control and minority norms conditions (both 55.6%) compared to the feedback and combined conditions (54.3% and 54.5%, respectively; χ^2^(3, N = 85,193) = 11.02, p = 0.012).

**Fig. 1:**
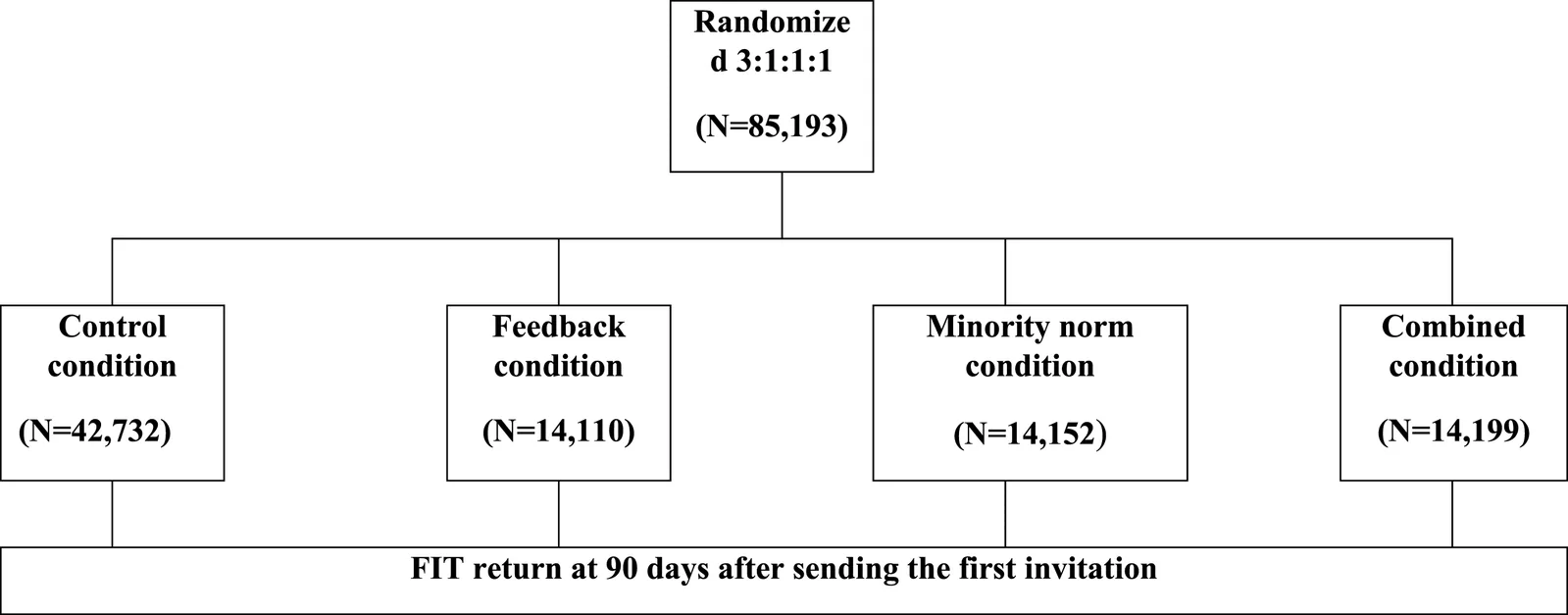
Study design.

**Table 2:** Socio-demographic characteristics of invited individuals (N = 85,193).

Variable	Control condition		Feedback condition		Minority norms condition		Combined condition		Total		p-value*
	N	%	N	%	N	%	N	%	N	%^b^	
Centre											
N-W Tuscany a	10072	50.3	3250	16.2	3361	16.8	3355	16.7	20038	23.5	0.099
Central Tuscany	17576	49.6	5960	16.8	5946	16.8	5940	16.8	35422	41.6	
S-E Tuscany a	15084	50.7	4900	16.5	4845	16.3	4904	16.5	29733	34.9	
Age (years)											
54-59	18953	49.7	6442	16.9	6281	16.5	6454	16.9	38130	44.8	0.012
60-70	23779	50.5	7668	16.3	7871	16.7	7745	16.5	47063	55.2	
Gender											
Female	20932	50.2	6947	16.7	6959	16.7	6834	16.4	41672	48.9	0.208
Male	21800	50.1	7163	16.5	7192	16.5	7365	16.9	43520	51.0	
Total	42732	50.2	14110	16.6	14152	16.6	14199	16.7	85193	100.0	

* P-value refers to Chi-Square test of independence.

a N-W Tuscany: North-Western; S-E: South-Eastern.

b Column percentage.

### 3.2 Screening uptake

Fig. 2 shows that the 90-day participation rate in the control group was 6.5% (2786/42,732). Participation was significantly higher in all three intervention groups: 8.4% in the feedback condition (1179/14,110), 7.4% in the minority norms condition (1042/14,152), and 7.7% in the combined condition (1086/14,199; χ^2^(3, N = 85,193) = 62.74, p < 0.001).

**Fig. 2:**
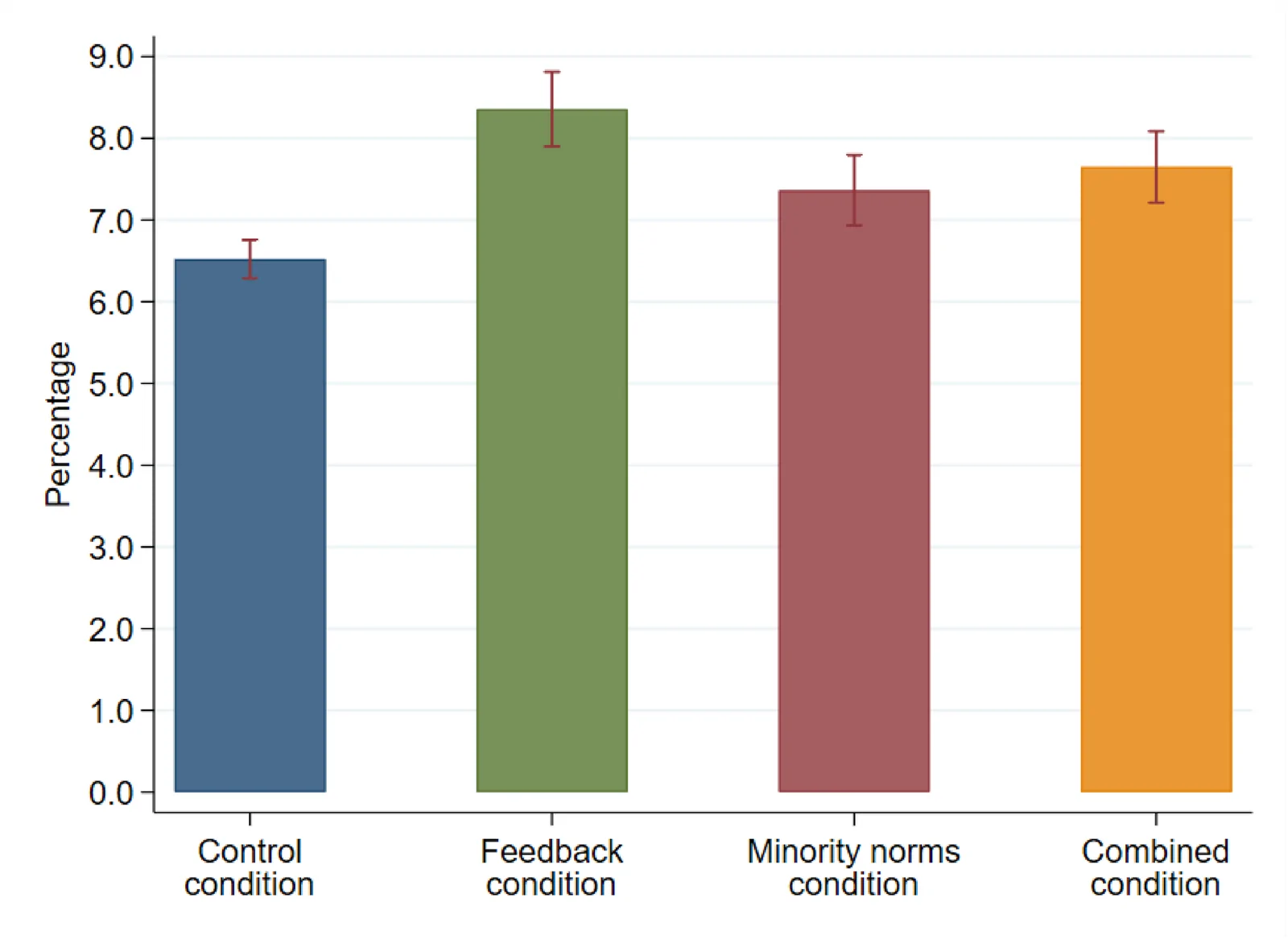
FIT return at 90 days from the first invitation letter across the experimental conditions.

A significant interaction between feedback and minority norms was detected in the 2×2 factorial analysis (OR: 0.80; 95%CI: 0.72-0.90; likelihood ratio test p-value = 0.0001), so results are presented by modality. In the mixed-effects logistic regression model, presented in Table 3, the feedback (adjusted odds ratio [aOR] 1.31, 95% CI 1.21–1.43, p < 0.001), minority norms (aOR 1.17, 95% CI 1.04–1.31, p = 0.011), and combined condition (aOR 1.22, 95% CI 1.05–1.43, p = 0.011) all increased participation compared with the control group. There were no significant differences between the three intervention conditions.

**Table 3:** Mixed effects logistic regression by age class. FIT return at 90 days.

	54-70 years					54-59 years					60-70 years				
	N	%	aOR^a^	95% CI	p-value	N	%	aOR^a^	95% CI	p-value	N	%	aOR^a^	95% CI	p-value
**Condition**															
Control condition	2786	6.5	ref			1352	7.1	ref			1434	6.0	ref		
Feedback condition	1179	8.4	1.31*	1.21-1.43	<0.001	558	8.7	1.21*	1.08-1.36	0.001	621	8.1	1.43*	1.28-1.60	<0.001
Minority norms condition	1042	7.4	1.17*	1.04-1.31	0.011	494	7.9	1.08	0.94-1.25	0.282	548	7.0	1.27*	1.10-1.48	0.001
Combined condition	1086	7.7	1.22*	1.05-1.43	0.011	484	7.5	1.02	0.85-1.22	0.847	602	7.8	1.49*	1.23-1.80	<0.001

**Gender**															
Male	2978	6.8	ref			1410	7.1	ref			1568	6.6	ref		
Female	3115	7.5	1.09*	1.04-1.15	<0.001	1478	8.1	1.14*	1.05-1.23	0.001	1637	7.0	1.05	0.98-1.13	0.181

**Age-class**															
54-59	2888	7.6	ref												
60-70	3205	6.8	0.88*	0.83-0.93	0.001										

*p < 0.05.

CI: confidence interval; aOR: adjusted odds ratio.

a Model with random intercept and random screening centre slope model.

Women were more likely to participate than men (aOR 1.09, 95% CI 1.04–1.15), and individuals aged 60–70 years were less likely to participate than those aged 54–59 years (aOR 0.88, 95% CI 0.83–0.93). Participation rates differed significantly across study centres (data not shown).

Subgroup analyses by age group showed that all three messages increased uptake among individuals aged 60–70 years (feedback aOR 1.43 (95% CI 1.28–1.60, p < 0.001); minority norms aOR 1.27 (95% CI 1.10–1.48, p = 0.001); combined aOR 1.49 (95% CI 1.23–1.80, p < 0.001). Among individuals aged 54–59 years, only feedback showed a significant effect (**aOR 1.21**, 95% CI 1.08–1.36, p = 0.001).

The best absolute risk reduction (ARR) and numbers needed to treat (NNT) were recorded for the feedback condition, respectively for all participants (54-70 years) 1.84% (95% CI 1.32%-2.35%), and 54 (95% CI 42-75); for participants aged 60-70 years 2.07% (95%CI: 1.39%-2.75%), and 48 (95% CI 36-71; see Table 4).

**Table 4:** Absolute Risk Reduction (ARR) and the Number Needed to Treat (NNT) by study arm for all participants (54-70 years) and for participants aged 60-70 years.

Arm	Overall (54-70 years)					60-70 years				
	Participation Rate (%)	ARR (%)^a^	95% C.I.	NNT^b^	95% C.I.	Participation Rate (%)	ARR (%)^a^	95% C.I.	NNT^b^	95% C.I.
Control	6.52	-	-	-	-	6.03	-	-	-	-
Feedback	8.36	1.84	1.32-2.35	54	42-75	8.10	2.07	1.39-2.75	48	36-71
Minority Norms	7.36	0.84	0.35-1.33	119	75-277	6.96	0.93	0.29-1.57	107	63-325
Combined condition	7.65	1.13	0.63-1.62	89	61-156	7.77	1.74	1.07-2.41	57	41-92

a ARR = Absolute risk reduction represents the difference between the participation rate in the control and experimental arms.

b NNT = Number needed to treat represents the average number of people who need to receive the intervention for one additional person to get screened. A lower NNT indicates a more effective intervention.

## 4 Discussion

This trial tested three cost-neutral, behaviourally informed modifications to invitation letters, feedback, minority-norms, and their combination, among previous non-responders across ten organised FIT screening programmes in Tuscany. In the overall population aged 54–70 years, all three interventions increased participation relative to the standard invitation. Feedback produced the largest absolute gains and the most favourable NNT, while minority-norms and combined messages showed clear benefits only in the older subgroup (60–70 years). Combining the two messages did not improve uptake beyond either message alone.

The prominent and consistent effect of the feedback message is consistent with the findings from the previous studies (Gorini et al., 2023, 2026). It also extends the evidence by demonstrating measurable increases from the social-norms and combined messages in a larger, more geographically diverse sample beyond Florence. At the same time, some age-specific patterns differ. Unlike in the first RCT, which found minority-norms to be effective across a wider middle-age band, here only feedback improved uptake among 54–59-year-olds and all messages were effective among 60-70-year-olds (Gorini et al., 2023). The results are therefore more in line with the second Florence RCT, where only feedback affected uptake among 54-59-year-olds and all messages had positive effects among older participants (Gorini et al., 2026).

These discrepancies are plausibly explained by differences in sample composition and setting: the current trial included rural programmes and a larger share of older non-attenders up to age 70, which may alter baseline norms and responsiveness to messaging. Despite these differences, the overall FIT return rate was similar to that observed previously, suggesting comparable absolute gains in re-engagement across studies.

The absence of an additive effect in the combined condition may reflect several plausible mechanisms, but this remains speculative because we did not collect process data on message perception or recall. One possibility is conceptual overlap, as the minority-norms message may have implicitly conveyed feedback by identifying recipients as non-participants, so adding an explicit feedback sentence provided little additional information. This hypothesis would be in line with prior evidence that feedback can drive behaviour across domains, including CRC screening, energy use and physical activity (Darby, 2006; Goldstein et al., 2008; Gorini et al., 2023, 2026; Normand, 2008), but it remains speculative. Alternatively, longer or more complex combined messages may have reduced attention or salience, or substitution or ceiling effects may have limited further gains. Effects could also be moderated by baseline norms or the age composition of the sample. These explanations are not mutually exclusive and cannot be confirmed here; future studies should include brief process measures (recall, perceived relevance, comprehension, and attention) and different study designs (factorial designs, message sequencing, or qualitative interviews) to distinguish among these mechanisms.

Unlike prior studies that communicated majority norms (Stoffel et al., 2019, 2021), our message combined information about peers' behaviour with data highlighting recipients’ minority status as non-participants in previous screening rounds. This positive effect corresponds with earlier research on energy consumption and physical activity (Darby, 2006; Normand, 2008).

The observed variations in FIT return rates across the ten Tuscany programmes are likely due to differences in programme features and implementation. The results show that providing feedback about prior behaviour or informing non-attenders of their minority status can increase FIT returns. They also highlight that these behaviourally informed messages are straightforward to implement and cost-neutral, as they only involve small changes to existing invitation letters (Gorini et al., 2023, 2026; Taylor et al., 2022).

The strengths of this study include its RCT design and the utilization of objective participation data. The factorial design allowed us to test for potential synergistic effects of combining different messages. Compared with the two previous trials, this study had a substantially larger sample and was conducted within a single region, which reduced variability in FIT return rates. Furthermore, the BE-inspired messages were co-developed through focus groups and specifically targeted individuals who had not participated in two consecutive CRC screening rounds. This high-risk group is challenging to engage, making even modest increases in uptake critical for screening efforts.

Furthermore, quantitative readability analyses using NLP techniques, together with crowd-sourced comprehension testing, showed that the modified invitation letters were as easy to understand as the standard invitation (Dell’Orletta et al., 2014).

The limitations of this study largely mirror those of the two preceding trials, since they were conducted in close succession using the same messages (Gorini et al., 2023, 2026). There was insufficient time between studies to address earlier constraints.

First, potential spill-over effects within households could not be controlled. Because non-attenders were individually randomised, members of the same household could have been allocated to different study arms. Second, the BE-inspired messages were included in the second paragraph of the invitation letter. Their salience might have been greater if placed at the start or highlighted with bold text or colour. Future studies could test these messages in shorter invitation letters and alternative placements. Third, the order of messages in the combined condition was not randomised, which may also have influenced results. Fourth, although we tested the combination of feedback and social-norms messages, the minority-norms message already contained an implicit element of feedback, limiting our ability to evaluate truly distinct combinations. Future research could explore combinations with more distinct interventions, such as the use of deadlines (Robb et al., 2025). Fifth, the statistical analysis was limited to age categories and gender. Future research should incorporate additional socioeconomic variables, such as deprivation, education, and health literacy, to better assess the effectiveness of BE messages. Additionally, such research should examine messages with more clearly separate components (for example, deadlines) and include brief process measures (recall, perceived relevance, comprehension, attention) to clarify mechanisms.

Regarding implications, this trial was conducted within organised, FIT-based screening programmes in Tuscany, which have moderate baseline uptake and a mix of long-standing and newer local programmes. Organised, population-based FIT programmes differ from opportunistic or colonoscopy-based systems and from settings with fragmented invitation processes, so the absolute impact of brief message changes may vary by screening model and baseline participation (Ola et al., 2024). Cultural norms, trust in health authorities, and programme maturity also influence how recipients respond to feedback and social-norms framing, so effects observed here are most directly applicable to similar organised FIT programmes and should be extrapolated to other contexts with caution (Le Bonniec et al., 2023; Stoffel et al., 2019, 2021).

## 5 Conclusions

This study extends prior research on BE-inspired messages by testing social-norms and feedback interventions in a new regional context. Feedback messages increased FIT return across all age groups, while minority-norms and combined messages showed age-specific effects. These findings highlight the potential of behavioural messages to improve screening participation and underscore the value of tailored communication strategies in population-based CRC screening programmes.

## CRediT authorship contribution statement

**Giuseppe Gorini:** Writing – review & editing, Writing – original draft, Visualization, Formal analysis. **Elisa Betti:** Writing – review & editing, Investigation, Formal analysis. **Patrizia Falini:** Writing – review & editing, Investigation. **Giulia Carreras:** Writing – review & editing, Investigation. **Beatrice Mallardi:** Writing – review & editing, Investigation. **Manola Migliori:** Writing – review & editing, Investigation. **Daniela Giorgi:** Writing – review & editing, Investigation. **Daniela Tramalloni:** Writing – review & editing, Investigation. **Alessandro Cosimi:** Writing – review & editing, Investigation. **Giulia Venturi:** Writing – review & editing, Investigation. **Andrea Amelio Ravelli:** Writing – review & editing, Investigation. **Anna Iossa:** Writing – review & editing, Investigation. **Francesca Battisti:** Writing – review & editing, Investigation, Conceptualization. **Sandro Tiziano Stoffel:** Writing – review & editing, Writing – original draft, Investigation, Conceptualization. **Paola Mantellini:** Writing – review & editing, Project administration, Investigation, Conceptualization.

## Ethics statement

The study protocol was approved by the Ethics Committees of the Local Health Units in Tuscany (Comitato Etico Toscana Area Vasta Centro, ID: 16875_spe, approved 19/01/21). Individual informed consent was not collected, as the trial was integrated into routine CRC screening, an Essential Level of Care (LEA) in the Italian National Health System, and it aimed to evaluate the effectiveness of the experimental invitation messages.

## Funding

This work was supported by the Tuscany Region [CUP F18D20000120002, 2019] through the BESTcc project.

## Declaration of competing interest

The authors declare that they have no known competing financial interests or personal relationships that could have appeared to influence the work reported in this paper.

## Data Availability

The statistical analysis and the individual de-identified data are available at: https://drive.google.com/drive/folders/1Jrhe18WKImyXhNk_J7Op_3GOdlb-wOfC?usp=sharing.
